# Novel therapeutic activities of dragon blood from palm tree *Daemonorops draco* for the treatment of chronic diabetic wounds

**DOI:** 10.1186/s40529-024-00422-2

**Published:** 2024-06-06

**Authors:** Hong-Chi Chen, Ren-In You, Fang-Mei Lin, Guan-Ling Lin, Tsung-Jung Ho, Hao-Ping Chen

**Affiliations:** 1 Department of Biomedical Sciences and Engineering, Tzu Chi University, Hualien, 970374 Taiwan; 2 Department of Laboratory Medicine and Biotechnology, Tzu Chi University, Hualien, 970374 Taiwan; 3 Department of Biochemistry, Tzu Chi University, 701, Sec 3, Zhongyang Road, Hualien City, 970374 Taiwan; 4 Integration Center of Traditional Chinese and Modern Medicine, Hualien Tzu Chi Hospital, 707, Sec. 3, Zhongyang Road, Hualien, 970473 Taiwan; 5 School of Post-Baccalaureate Chinese Medicine, Tzu Chi University, Hualien, 970374 Taiwan; 6 Department of Chinese Medicine, Hualien Tzu Chi Hospital, Hualien, 970473 Taiwan

**Keywords:** Dragon blood, Myoblast differentiation, Plasminogen activator inhibitor, Stem cell proliferation, Wound healing

## Abstract

**Background:**

The clinical efficacy of Jinchuang Ointment, a traditional Chinese medicine (TCM), in treating chronic non-healing diabetic wounds has been demonstrated over the past decades. Both in vitro and in vivo angiogenic activities have been reported for its herbal ingredients, including dragon blood from the palm tree *Daemonorops draco* and catechu from *Uncaria gambir* Roxb. Additionally, crude extracts of dragon blood have exhibited hypoglycemic effects not only in animal studies but also in cell-based in vitro assays.

**Results:**

Our findings indicate that crude dragon blood extract promotes the differentiation of myoblasts into myotubes. Partially purified fractions of dragon blood crude extract significantly enhance the expression of muscle cell differentiation-related genes such as *myoG*, *myoD*, and *myoHC*. Our results also demonstrate that crude extracts of dragon blood can inhibit platelet-derived growth factor-induced PAI-1 expression in primary rat vascular smooth muscle cells, thereby favoring changes in hemostasis towards fibrinolysis. Consistent with previous reports, reduced expression of plasminogen activator inhibitor 1 (PAI-1) accelerates wound healing. However, further separation resulted in a significant loss of both activities, indicating the involvement of more than one compound in these processes. Stem cells play a crucial role in muscle injury repair. Neither dragon blood nor catechu alone stimulated the proliferation of human telomerase reverse transcriptase (hTERT)-immortalized and umbilical cord mesenchymal stem cells. Interestingly, the proliferation of both types of stem cells was observed when crude extracts of dragon blood and catechu were present together in the stem cell growth medium.

**Conclusions:**

Dragon blood from *D. draco* offers multifaceted therapeutic benefits for treating chronic nonhealing diabetic wounds from various perspectives. Most drugs in Western medicine consist of small molecules with defined ingredients. However, this is not the case in TCM, as the activities of dragon blood reported in this study. Surprisingly, the activities documented here align with descriptions in ancient Chinese medical texts dating back to A.D. 1625.

## Introduction

For decades, Jinchuang ointment, a traditional Chinese medicine (TCM), has been effectively utilized in Taiwan to treat chronic non-healing diabetic (Ho et al. 2016, 2020) and leprosy wounds (Hsu et al., 2019). Several years ago, amused and motivated to understand the mechanism of action behind this folk remedy, we delved into the biological activities of its herbal components. Notably, the in vivo and in vitro wound healing activity of dragon blood has been reported previously (Ji et al. 2015; Lu et al. 2021). Moreover, dragon blood and catechu have exhibited potent in vivo and in vitro angiogenic activity, which is pivotal in the wound healing (Ho et al. 2022; Krishnaraj et al. 2019). Dragon blood, a red resin obtained from the fruit of tropical palm tree *Daemonorops draco* found in Indonesia and Malaysia (Wu et al. 2019), has recently demonstrated a hypoglycemic effect in animal studies (Ching et al. 2023).

In the commentary on the ancient Chinese medical book, Shennong Ben Cao Jing Shu (神農本草經疏), published during the Ming Dynasty (A.D. 1625), the efficacy of dragon blood is described as “dispelling stagnant blood and promoting the growth of fresh tissue in wounds” (破積血, 金瘡生肉). Inspired by this description, we examined the biological activity of crude extracts of dragon blood using various in vitro assays. Our investigation encompassed platforms for the differentiation of myoblasts into multinucleated myotubes, regulation of fibrinolysis, and proliferation of stem cells.

**Fig. 1:**
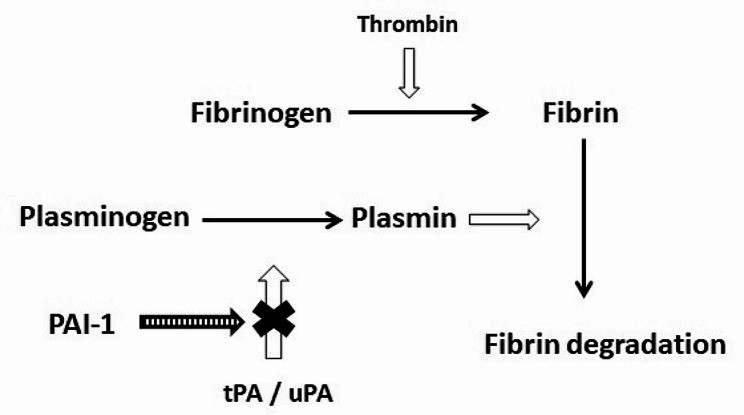
Role of PAI-1 in the fibrinolytic system

Plasmin, derived from plasminogen by digestion with tissue plasminogen activator (tPA) or urokinase plasminogen activator (uPA), is a protease that degrades fibrin clots (Fig. 1). Plasminogen activator inhibitor-type 1 (PAI-1) negatively regulates fibrinolysis by binding to plasminogen activators, thus inhibiting tPA and uPA activities. PAI-1, primarily stored in the α-granules and released upon platelet activation (Brogren et al. 2011), is also secreted from endothelial cells, macrophages, fibroblasts, and vascular smooth muscle cells. Elevated plasma levels of PAI-1, indicating the decrease of decreased fibrin degradation, can lead to pathological fibrin accumulation and is a risk of thrombosis (Morrow and Mutch, 2022). Therefore, increased plasma PAI-1 activity is considered a cardiovascular risk factor (Nordt et al. 1999). Additionally, PAI-1 is implicated in skeletal muscle regeneration (Rahman and Krause 2020) and cancer progression and metastasis (Kubala and DeClerck 2019). While platelets are the main storage location for platelet-derived growth factor (PDGF), the expression of PAI-1 in vascular smooth muscle cells can be stimulated by PDGF, the first growth factor identified and discovered in platelets (Heldin 2003; Reilly and McFall 1991). Hence, the effects of crude dragon blood extract on PDGF-induced PAI-1 expression were investigated.

Mesenchymal stem cells promote angiogenesis and reepithelialization, participate in immune regulation, mitigate inflammation, and ultimately facilitate repair of diabetic foot ulcers (Yu et al. 2023). In response to injury, skeletal muscle stem cells undergo myogenesis, proliferate rapidly, differentiate, and fuse to form multinucleated myotubes (Thakur et al. 2020). To gain deeper insight into the role of dragon blood in diabetic wound healing, we examined its effects on myoblast differentiation, PAI-1-mediated fibrinolysis regulation, and stem cell proliferation. In this context, we present, for the first time, the biological activities of dragon blood from *D. draco* in the treatment of chronic diabetic wounds.

## Methods

### Materials

Molecular biology-grade dimethyl sulfoxide (DMSO) was procured from Sigma-Aldrich (St. Louis, MO, USA). Platelet-derived growth factor-BB (PDGF-BB) (Catalog number: 100-14B) was obtained from PeproTech (Rehovot, Israel). 4’,6-Diamidino-2-phenylindole dihydrochloride (DAPI) was purchased from Invitrogen™ (Waltham, MA, USA). Silica gel 60 (230–400 mesh) was sourced from Acme Synthetic Chemicals (Mumbai, India). Dragon blood, a resin obtained from the fruit of *Daemonorops draco* (Wu et al. 2019), and catechu, a dried, evaporated decoction of *Uncaria gambir* Roxb (Ho et al. 2022), have been previously documented (Ching et al. 2023; Ho et al. 2022).

### Preparation of catechu and dragon blood crude extracts

Herbal raw materials, including catechu and dragon blood samples, were collected and finely powdered (1 g). The resulting powder was dissolved in methanol (10 mL). After sonication for 20 min and overnight soaking, centrifugation at 4500 × g for 5 min was performed to remove undissolved particles. The supernatant was then dried overnight in a Savant SpeedVac™ vacuum concentrator (Thermo Fisher Scientific Inc., Waltham, Massachusetts, USA). The resulting dry catechu and dragon blood crude extracts were dissolved in DMSO (0.1 g/mL), and the DMSO stock solution was utilized for subsequent in vitro experiments.

### Effects on C2C12 myoblast differentiation into myotubes

The source and culture conditions for murine skeletal muscle cell lines (C2C12 myoblasts) and primary rat aortic smooth muscle cells (RASMC) have been previously documented (Feener et al. 1995; Huang et al. 2015). C2C12 cells (4 × 10^5^) were seeded in a 6 cm culture dish. Upon reaching 70 − 80% confluency, the cell culture medium was replaced with a medium containing 3% horse serum to initiate myoblast differentiation. Subsequently, crude extracts (0.1 ng/mL) of catechu or dragon blood were added. The culture medium was refreshed daily. Cells were fixed with methanol on day 6 (Huang et al. 2015). Myosin heavy chain (MHC) was detected using a monoclonal antibody (1:50; Abcam Limited, Cambridge, UK), followed by a fluorescence-conjugated secondary antibody (1:1000; Jackson ImmunoResearch Laboratories Inc., West Grove, PA, USA). Cell nuclei were counterstained with DAPI. Immunofluorescent images were acquired using a confocal microscope (Nikon C2si + System; Tokyo, Japan).

To further isolate the active compounds facilitating myoblast differentiation, dragon blood (5 g) was extracted with 10 mL methanol as described previously. Methanol was then evaporated *in vacuo*. Dry crude extracts of dragon blood were fractionated using a silica gel column (2.6 × 35 cm) packed with 60 g silica gel. The column was isocratically eluted with four solvents: Solvent 1 (600 mL) (*n*-hexane: ethyl acetate; methanol = 6:1:0), Solvent 2 (600 mL) (*n*-hexane: ethyl acetate; methanol = 0:1:0), Solvent 3 (600 mL) (*n*-hexane: ethyl acetate; methanol = 0:6:1), and Solvent 4 (400 mL) (*n*-hexane: ethyl acetate; methanol = 0:0:1). Dracorhodin, an indicator standard compound found in *D. draco* blood, eluted with solvent 1. Fractions containing dracorhodin were designated as fraction 1, while fractions after its elution were termed fraction 2. Fractions 3, 4, and 5 were eluted with solvents 2, 3, and 4, respectively. Five primary fractions were obtained and divided into ten aliquots each. The solvent was then evaporated *in vacuo*, and the dry solids in each aliquot were dissolved in 0.5 mL DMSO and stored at − 80 °C.

Partially purified dragon blood DMSO stocks were added to the myoblast differentiation medium (600-fold and 6000-fold dilutions). Cell samples were collected after 72 h of differentiation. Experimental procedures for RNA extraction and qRT-PCR were conducted using an RNeasy Plus Kit (Qiagen, Hilden, Germany) and an Applied Biosystems 7500 Real-Time System (Waltham, MA, USA), respectively, with SYBR Green Real-Time PCR Master Mix (ThermoFisher, Waltham, MA, USA). The relative expression of three muscle cell differentiation-related genes, myoblast determination protein (*myoD*), myogenin (*myoG*), and myosin heavy chain (*myHC*), was calculated using Applied Biosystems 7500 software. All primer pairs were obtained from PrimerBank or designed using Primer Express software (Life Technologies, Carlsbad, CA, USA) (Huang et al. 2015).

### Effects of dragon blood on mesenchymal stem cell proliferation

Human telomerase reverse transcriptase (hTERT)-immortalized mesenchymal stem cells from cord blood (cbMSC-hTERT, BCRC 60,604) were purchased from the Bioresources Collection and Research Center (Hsinchu, Taiwan) (Yao and Hwang 2012). The cbMSC-hTERT cells were cultured in minimum essential medium supplemented with 20% fetal bovine serum, 0.1 mM non-essential amino acids (Gibco, Carlsbad, CA, USA), 1.0 mM sodium pyruvate (Gibco, Carlsbad, CA, USA), 4 ng/mL human-bFGF (Gibco, Carlsbad, CA, USA), and 30 µg/mL Hygromycin B (Gibco, Carlsbad, CA, USA). Culturing was carried out in a humidified atmosphere containing 5% CO_2_ at 37 °C.

Human umbilical cord samples were obtained from the Department of Obstetrics and Gynecology, Mennonite Christian Hospital, Hualien, Taiwan. These samples were cut into small pieces and treated with 1 mg/mL collagenase IA (Sigma-Aldrich, Saint Louis, MO, USA) at 37 °C for 18 h. The resulting mixture was washed with PBS and digested with 0.5% trypsin at 37 °C for 30 min. Trypsin was neutralized using DMEM medium containing 10% FBS, and the cells were centrifuged at 300 × g for 10 min. The cells were then resuspended in DMEM medium supplemented with 10% FBS, 2 mM L-glutamine (Gibco, Carlsbad, CA, USA), and 100 U/mL Penicillin/10 µg/mL Streptomycin (Gibco, Carlsbad, CA, USA) and cultured at 37 °C, 5% CO_2_ in a humidified atmosphere. Adherent primary umbilical cord mesenchymal stem cells (UCMSCs) were cultured until they reached 80% confluence for further passages (Fu et al. 2006). Third- to fifth-passage UCMSCs were characterized by flow cytometry using CD29-PE, CD44-APC, CD105-APC, and CD34-FITC antibodies. MSCs express CD29, CD44, and CD105 and are negative for CD34 (Sober et al. 2023).

Cell viability was assessed using the Cell Counting Kit-8 (Merck KGaA, Darmstadt, Germany) following the manufacturer’s instructions. Briefly, 5 × 10^3^ cbMSC-hTERT cells or 3 × 10^3^ UCMSCs were seeded in each well of a 96-well culture plate and cultured overnight. The cells were then treated with gradient concentrations of Dragon’s blood and Catechu extract (0–50 µg/mL) for 48 h. After incubation, the cells were treated with 10 µL of CCK-8 solution in each well for 2–3 h. The optical absorbance at 450 nm was measured using a CLIARIOstar® Plus Microplate Reader (BMG Labtech, Cambridge, MA, USA).

### Effect on the expression of *PAI-1* gene and PAI-1 proteins induced by PDGF-BB

Upon reaching 100% confluence, VSMC were starved overnight in DMEM-L culture medium supplemented with 0.1% BSA. To assess PAI-1 mRNA expression, cells were pre-treated with crude dragon blood extract for 10 min before stimulation with PDGF-BB for 3 h. RNA extraction was then performed using the Gene-spin Total RNA Purification Kit (Protech Technology Enterprise Co., Ltd., Taipei, Taiwan), followed by reverse transcription using the MMLV Reverse Transcription Kit (Protech Technology Enterprise Co., Ltd., Taipei, Taiwan). All experimental procedures strictly followed the manufacturer’s instructions. The *36B4* gene served as an internal control gene in the quantitative PCR (qPCR) experiment. Primer sequences used in this study are detailed in Table 1. Additionally, to evaluate PAI-1 protein production, cells were stimulated with PDGF-BB for 24 h, and the conditioned medium was collected for Western blot analysis.

**Table 1:** Primer sequences for RT-qPCR of target genes

Gene	Primer Sequence
Rat *PAI-1*	Forward: GCACGAGTACGACATCCTGG
	Reverse: GCCCTCTGAGGTCCACTTCA
Rat *36B4*	Forward: CCGTGTGAGGTCACAGTACC
	Reverse: GTAGTCAGTCTCCACAGACAAAGC
Mouse *myoG*	Forward: CAGTACATTGAGCGCCTACA
	Reverse: GGACCGAACTCCAGTGCAT
Mouse *myoD*	Forward: GCTGCCTTCTACGCACCTG
	Reverse: GCCGCTGTAATCCATCATGC
Mouse *myHC*	Forward: CTCAAGGAATCCCGGTCCTT
	Reverse: TTCTCGGCAATCTGTTCAGTGA
Mouse β-actin	Forward: AGAGCTACGAGCTGC
	Reverse: AGCACTGTGTTGGCGTACA

## Results

### Effects of dragon blood on C2C12 myoblast differentiation into myotubes

**Fig. 2:**
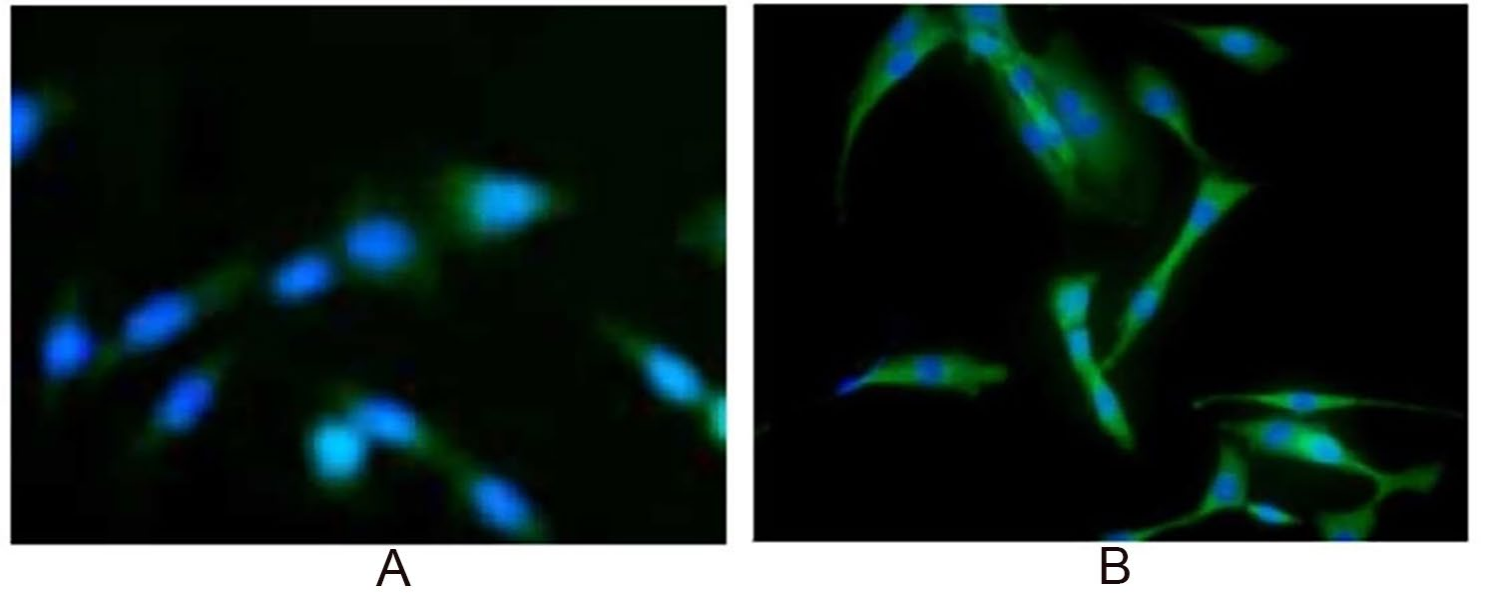
Immunostaining of C2C12 cells with MHC antibody (green) in the presence of herbal crude extracts (**A**) Catechu and (**B**) Dragon blood. C2C12 cells were cultured for 6 days with partially purified crude extracts of dragon blood. Myotubes were immunofluorescently labeled with an anti-MHC monoclonal antibody (green) and counterstained with DAPI for nuclei (blue)

Muscle fibers typically regenerate following injury-induced necrosis (Laumonier and Menetrey 2016). Satellite cells, located beneath the basal lamina surrounding muscle fibers, play a crucial role in this regenerative process (Bachman and Chakkalakal 2022). The fusion of precursor myoblasts contributes to the formation of multinucleated muscle fibers and myotubes. To explore whether the primary herbal components of “Jinchuang ointment” facilitate myoblast differentiation, we employed an in vitro differentiation model. C2C12 myoblasts were treated with crude extracts of catechu and dragon blood in a medium containing 3% horse serum to initiate differentiation. Immunostaining for myosin heavy chains on day 6 revealed that only crude dragon blood extracts promoted the formation of myotubes through primary myoblast fusion (Fig. 2).

**Fig. 3:**
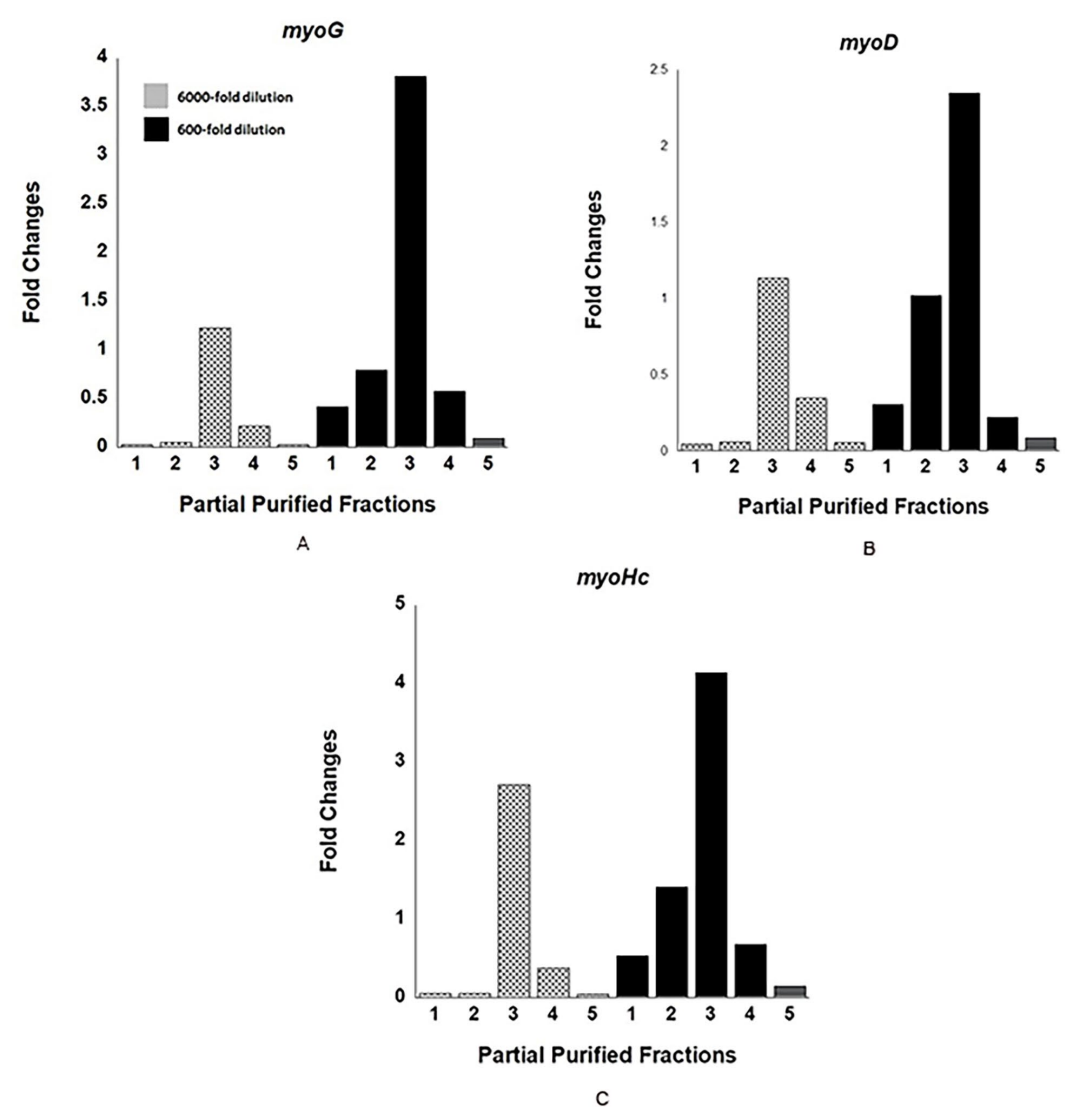
Effects of partially purified dragon blood fractions on muscle cell differentiation-related genes **(A)** *myoG*, **(B)** *myoD*, and **(C)** *myoHC*. Crude extracts of dragon blood were separated by isocratic silica gel chromatography into several fractions. Fraction 1: 600 ml, *n*-hexane: ethyl acetate = 6:1 (dracorhodin-containing fractions); Fraction 2: 600 ml, *n*-hexane: ethyl acetate = 6:1 (fractions after dracorhodin elution); Fraction 3: 600 ml, ethyl acetate; Fraction 4: 600 ml, ethyl acetate: methanol = 6:1; Fraction 5: 400 ml, methanol

To isolate the active compounds responsible for myoblast differentiation facilitation, crude dragon blood extracts underwent isocratic purification via silica gel chromatography. Four different solvents were sequentially used to elute the compounds (Solvent 1: 600 mL, *n*-hexane: ethyl acetate = 6:1; Solvent 2: 600 mL, ethyl acetate; Solvent 3: 600 mL, ethyl acetate: methanol = 6:1; Solvent 4: 400 mL, methanol). The ability of each fraction to stimulate the expression of three muscle cell differentiation-related genes (*myoD*, *myoG*, *myHC*) was assessed using RT-qPCR. As depicted in Fig. 3, expression levels of all three genes were significantly elevated in fraction 3 after 48 h. However, further separation of compounds within fraction 3 by HPLC resulted in a substantial loss of activity for all three genes (data not shown), suggesting the involvement of multiple different active compounds within fraction 3. Once they were separated, the activity got lost. The purification and identification of these molecules may pose challenges and require meticulous efforts.

### Effects of dragon blood on mesenchymal stem cell proliferation

**Fig. 4:**
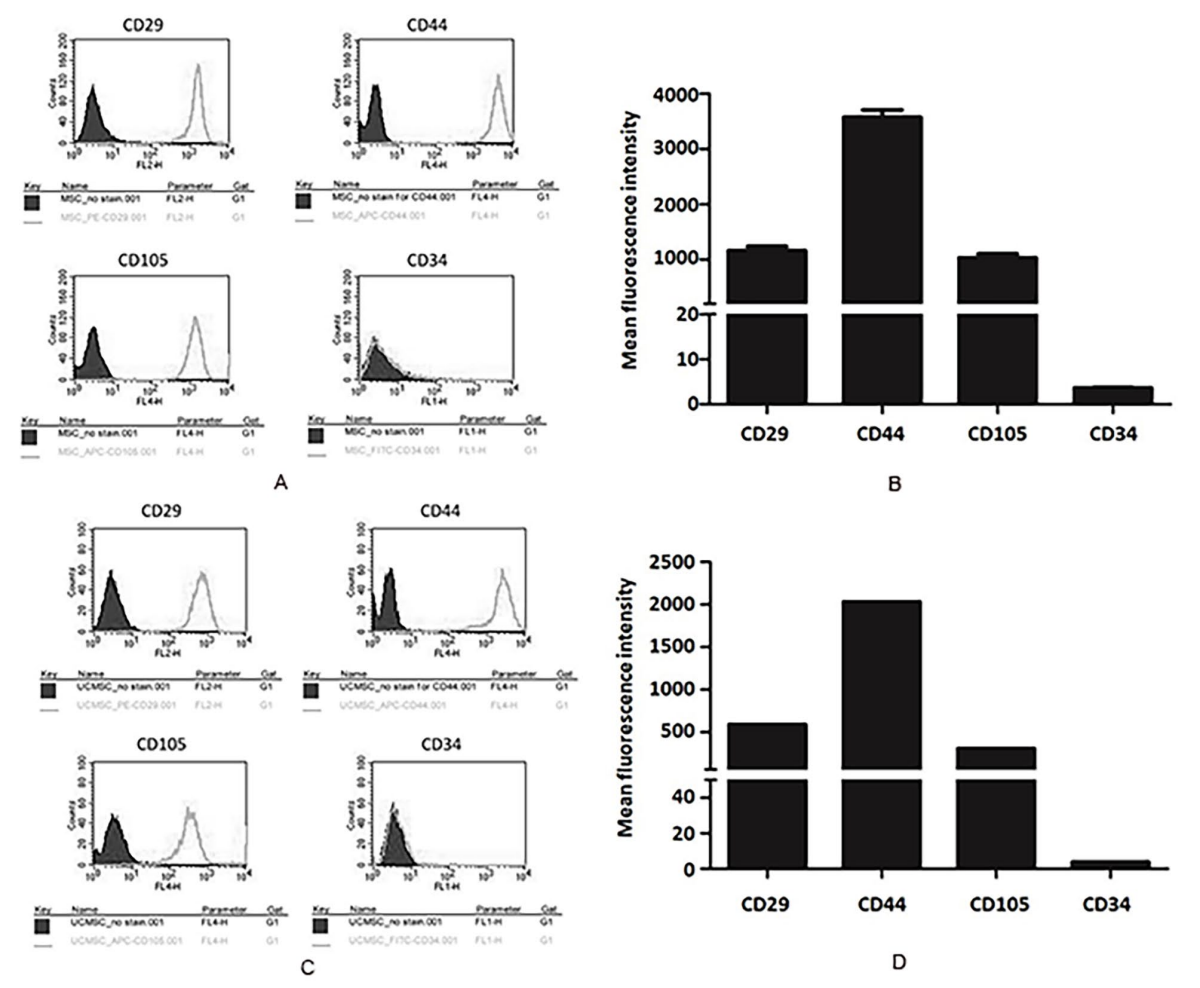
Characterization of cbMSC-hTERT and UCMSCs using flow cytometry. **(A, B)** cbMSC-hTERT and **(C, D)** isolated UCMSCs were stained with PE-CD29, APC-CD44, APC-CD105, and FITC-CD34 stained (▁), compared to unstained (■) and analyzed by flow cytometry. The cells were positive for CD29, CD44, and CD105, and negative for CD34

Stem cells, also known as satellite cells, play a pivotal role in repairing muscle injuries (Bachman and Chakkalakal 2022). Therefore, investigating whether the herbal components present in “Jinchuang ointment” exhibit stem cell activity is of significant interest. Commercially available cbMSC-hTERT and UCMSCs isolated from umbilical cord tissue were characterized using flow cytometry, serving as a model to assess the activity of the herbal materials in this study. Both cells exhibited strong positivity for MSC surface epitopes, including CD29, CD44, and CD105, but showed no positivity for the hematopoietic stem cell marker CD34 (Fig. 4), consistent with previous reports (Sober et al. 2023).

**Fig. 5:**
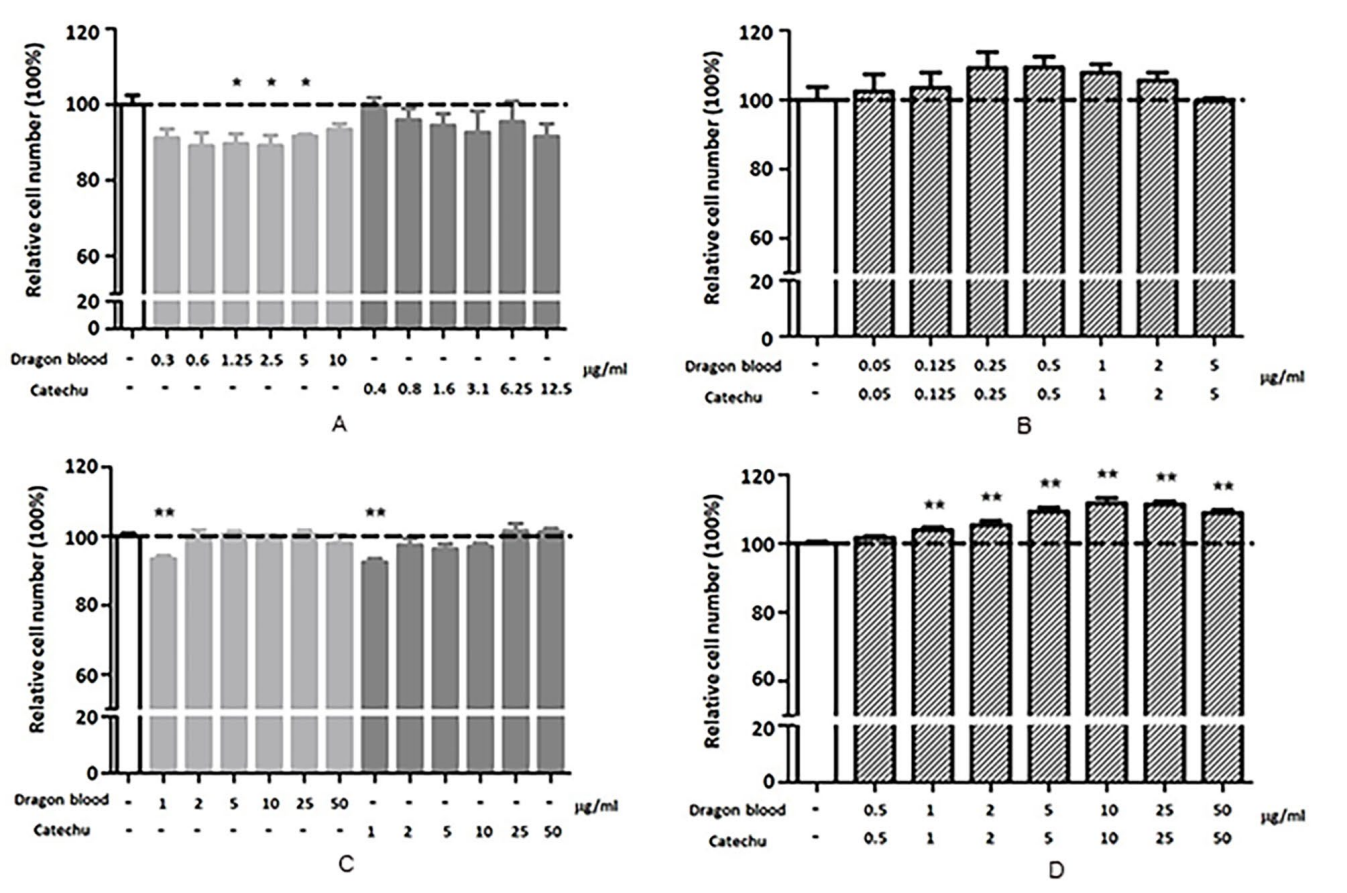
Combination of dragon blood and catechu extract promotes the proliferation of UCMSCs. **(A, B)** Human cbMSC-hTERT cells and **(C, D)** UCMSCs were treated with various concentrations of Dragon’s blood extract, Catechu extract, or a combination of both for 48 h. Cell viability was assessed using a CCK-8 assay (*n* = 3–4). **P < 0.01 compared to the vehicle control

To evaluate the cytotoxicity and effects of crude herbal extracts on human stem cells, cell viability assay was performed. Neither cytotoxicity nor stimulation of stem cell proliferation was observed when stem cells were treated with crude extracts of catechu (up to 50 µg/mL) or dragon blood (up to 100 µg/mL) alone for 48 h (Data not shown). However, it is noteworthy that the growth of cbMSC-hTERT and UCMSCs was enhanced only in the presence of both dragon blood and catechu crude extracts (Fig. 5). Intriguingly, the interaction between the two different crude herbal extracts, rather than individual herbal extracts, appeared to stimulate the proliferation of these stem cells. Consequently, identifying the active compounds involved in this process presents a significant challenge.

### Effects of dragon blood on the expression of *PAI-1* gene and secretion of PAI-1 proteins induced by PDGF-BB

**Fig. 6:**
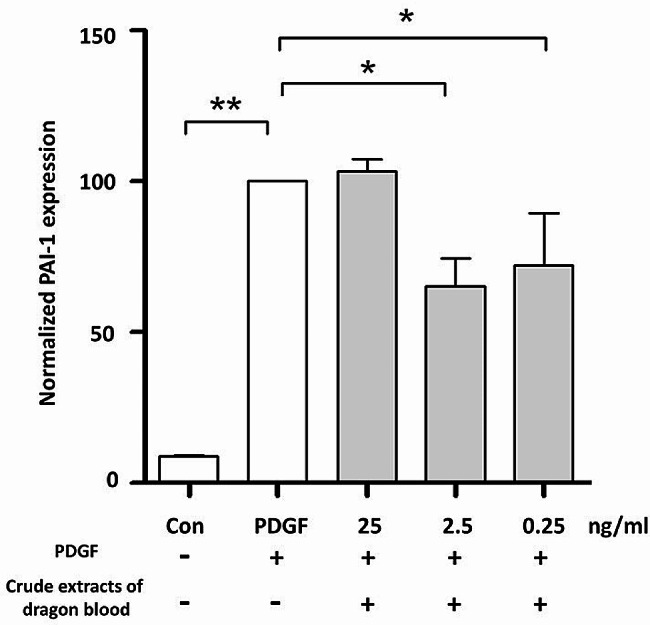
Crude extracts of dragon blood decrease *PAI-1* Gene Expression in VSMCs. One-way analysis of variance (ANOVA) was conducted for statistical analysis (*n* = 3). Duncan’s test was used for post hoc analysis. (*P < 0.05, **P < 0.001)

**Fig. 7:**
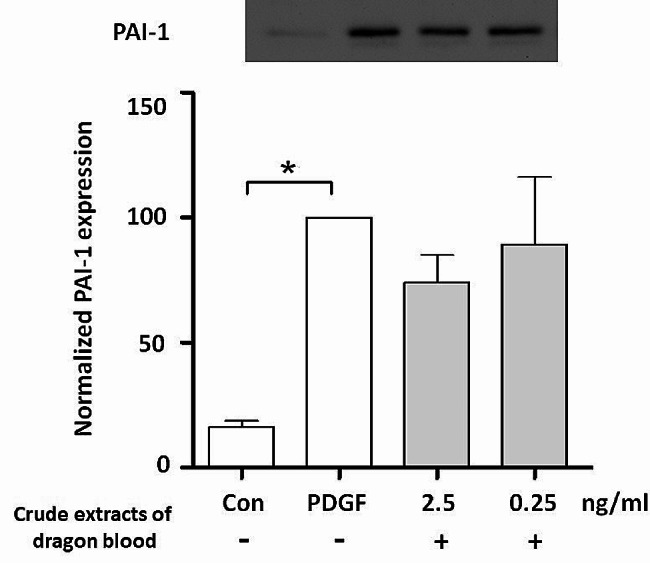
Crude extracts of dragon blood decrease the secretion of PAI-1 proteins. One-way analysis of variance (ANOVA) was conducted for statistical analysis (*n* = 3). Tukey test was used for post hoc analysis. (*P < 0.05)

Blood stasis removal is a prominent efficacy attributed to dragon blood in TCM. Therefore, we evaluated the impact of crude dragon blood extract on the expression of the *PAI-1* gene and secretion of PAI-1 proteins in VSMCs. qPCR results revealed a significant increase in *PAI-1* expression in the presence of PDGF-BB, and the addition of 2.5 ng/mL and 0.25 ng/mL crude dragon blood extract led to a notable decrease in PAI-1 mRNA expression by 65.19% ± 15.95% and 72.05% ± 30.02%, respectively (Fig. 6). The secretion of PAI-1 protein into the conditioned medium was assessed via Western blotting. Consistently, treatment with 2.5 ng/mL crude dragon blood extracts resulted in a reduction of PAI-1 secretion by 73.79% ± 18.94% (Fig. 7).

## Discussion

Wound healing typically progresses through four phases: [i] bleeding and hemostasis, [ii] inflammation, [iii] proliferation, and [iv] remodeling. In patients with diabetes, the chronic non-healing wounds are a common occurrence and often detained in the inflammation and proliferation phases (Brem et al. 2007). The underlying physiological factors contributing to these impairments are multifaceted and encompass poor angiogenic activity, altered expression of growth factors and cytokines, reduced migration of fibroblasts and keratinocytes, prolonged presence of neutrophils and macrophages, and diminished formation of granulation tissue (Brem et al. 2007). Previous studies have highlighted the potential of dragon blood from the palm tree *D. draco* to stimulate both in vitro and in vivo angiogenesis (Krishnaraj et al. 2019; Li et al. 2016), along with the migration of fibroblasts and keratinocytes (Jiang et al. 2018; Lu et al. 2021). Additionally, mesenchymal stem cells have been recognized for their role in promoting angiogenesis, re-epithelialization, inflammation reduction, and the repair of diabetic foot ulcers (Yu et al. 2023). Our findings demonstrate that the combined use of crude extracts of dragon blood and catechu promotes the proliferation of mesenchymal stem cells, suggesting the rationality of employing “Jinchuang Ointment,” which contains both catechu and dragon blood, in the treatment of chronic non-healing diabetic wounds.

Elevated levels of PAI-1 have been observed in diabetic patients (El Sewy et al. 2022; Lyon and Hsueh 2003), and PAI-1 levels are higher than non-diabetic control in the arterial wall from people with type II diabetes (Pandolfi et al. 2001). Studies have shown accelerated skin wound healing in PAI-1-deficient (PAI-1-/-) mice (Chan et al. 2001). On the other hand, the expression PAI-1 was shown to have negative effects on vascular wound healing and arterial neointima formation in mouse injury models (Carmeliet et al. 1997). Consistently, the application of compound PAI-039, an inhibitor of PAI-1, has been found to enhance dermal wound closure in diabetic patients (Rebalka et al. 2015), but topically treated PAI-1 antagonist tiplaxtinin results in a decreases in wound closure and re-epithelialization (Simone et al. 2015). Our study indicates that crude extracts of dragon blood from *D. draco* reduce the expression of PAI-1. Considering the dynamic, if not complicated, involvement of PAI-1 in various stages of skeletal muscle regeneration, an essential aspect of wound healing (Rahman and Krause 2020), the potential therapeutic benefit of regulating PAI-1 expression in treating chronic diabetic wounds warrants attention.

Among the array of factors influencing PAI-1 expression, the role of PDGF is noteworthy. As the first identified growth factor, PDGF plays significant roles in embryonic development, cell proliferation, migration, and angiogenesis. PDGF not only stimulates VSMC proliferation (Millette et al. 2005) but also promotes phenotypic states changes from contractile to synthetic in VSMCs (Holycross et al. 1992). While PDGF-BB has been implicated in accelerating the wound healing process (Pierce et al. 1991), its overexpression has been associated with various diseases, including atherosclerosis, fibrotic disorders, and malignancies. The activation of the PDGF receptor and the downstream signaling pathways have been implicated in the development of type II and gestational diabetes mellitus, affecting liver and pancreas islet β cells at least, and diabetic complications, including insulin resistance, diabetic wound healing and atherosclerosis (Cao et al. 2023; Shen et al. 2020). Furthermore, elevated serum PDGF levels have been reported in diabetic adults (Josefsberg et al. 1992), and are positively correlated with diabetes progression stages (Chen 2019), suggesting a potential link between increased serum PDGF-BB levels in diabetic patients, enhanced PAI-1 expression in VSMCs, and the complexity of chronic diabetic wounds.

Using pharmacological inhibition, Leik et al. demonstrated the involvement of PAI-1 in in vivo angiogenesis and smooth muscle cell (SMC) adhesion and migration, contributing to tissue remodeling (Leik et al. 2006). Notably, PAI-1, acting through the low density lipoprotein receptor-related protein-1 (LRP1) (Degryse et al. 2004), was shown to increase the expression of vitronectin in VSMCs (Luo et al. 2017), implicating its autocrine role in vascular remodeling. Furthermore, a study conducted on patients with type 2 diabetes revealed that PAI-1 expression was augmented in the plasma and subcutaneous adipose tissue of both type 2 diabetes and obese groups (Mossberg et al. 2022), suggesting that PAI-1 secreted from various cells or tissues may, collectively, adversely affect the healing of chronic diabetic wounds. Given the multifunctional roles of PAI-1 in both physiological and pathological aspects, the effects of dragon blood on PDGF-BB-induced PAI-1 expression in different cell types and tissues during wound healing would be interesting, but difficult, to be explored.

VPS39 serves as an important regulator of myoblast differentiation and muscle glucose uptake. Reduced VPS39 expression in myoblasts and myotubes has been observed in patients with type 2 diabetes (Davegårdh et al. 2021). More recently, our group reported the stimulation of in vitro and in vivo glucose uptake by dragon blood from *D. draco* (Ching et al. 2023). Additionally, the results of our study demonstrated that dragon blood stimulated myoblast differentiation into myotubes. In short, dragon blood from *D. draco* offers multifaceted therapeutic benefits for treating chronic non-healing diabetic wounds.

Medication cost is a significant concern for patients. Due to the lack of a patent for the use of this folk medicine, the price of dragon blood is much lower than that of most Western medicines. While most drugs used in Western medicine consist of defined ingredients, the herbal components used in traditional Chinese medicine (TCM) are often complex, and many of them are unknown, similar to the activities of dragon blood reported in this study. Surprisingly, the activities reported in this study align well with the descriptions found in ancient Chinese medical books published in A.D. 1625.

## Data Availability

Data and materials will be accessible upon request.
